# Account of Diseases in an Eastern District of London, from the 20th of August to the 20th of September 1807

**Published:** 1807-10

**Authors:** 


*Account of Diseases in an Eastern District of London, from the 20th of August to the 20th of September* 1807.

| | |
|---|---|
| ACUTE DISEASES. | |
| Scarlatina Anginosa - | 4 |
| Hæmorrhagia Intestinalis | 1 |
| Icterus - - - | 1 |
| Cholera - - - | 12 |
| CHRONIC DISEASES. | |
| Tussis - - - | 12 |
| Dyspnœa - - - | 5 |
| Tussis & Dyspnœa - | 10 |
| Hydrothorax - - | 2 |
| Anasarca - - - | 3 |
| Enterodynia - - | 5 |
| Dysenteria - - | 1 |
| Diarrhœa - - - | 7 |
| Herpes - - - | 3 |
| Scrophula - - | 2 |
| Hysteria - - - | 2 |
| Amenorrhœa - - | 7 |
| Menorrhagia - - | 3 |
| Fluor Albus - - | 5 |
| Hypochondriasis - | 2 |
| Dyspepsia - - | 4 |
| Rheumatismus Chronicus | 11 |
| PUERPERAL DISEASES. | |
| Dolores Post Partum - | 4 |
| Mammæ Abscessus - | 2 |
| Rhagas Papillæ - | 4 |
| INFANTILE DISEASES. | |
| Febris Mesenterica - | 2 |
| Ophthalmia - - | 5 |
| Aphthæ - - - | 4 |
| Vermes - - - | 5 |

The disease which formed a principal part of the list of the last month, has been protracted to the present list, Cholera still prevails to a considerable degree. The evacuation per anum has, in several instances, been much less than in former cases; but the pain preceding it has been greater, and the disorder has yielded more readily to the use of aperient medicines, than to opiates and astringents. The griping pains complained of, may undoubtedly, in many instances, be attributed to the too early check that has been given to the evacuation.

